# Synthesis of a paraffin phase change material microencapsulated in a siloxane polymer

**DOI:** 10.1007/s00396-012-2782-z

**Published:** 2012-09-07

**Authors:** Witold Fortuniak, Stanislaw Slomkowski, Julian Chojnowski, Jan Kurjata, Adam Tracz, Urszula Mizerska

**Affiliations:** Center of Molecular and Macromolecular Studies, Polish Academy of Sciences, Sienkiewicza 112, 90-363 Łódź, Poland

**Keywords:** PCM microcapsules, Microencapsulation, Microencapsulated phase change materials, Polyhydromethylsiloxane, Microcapsules, Karstedt catalyst

## Abstract

The coemulsification method suitable for the formulation of microcapsules of *n*-eicosane coated with a polysiloxane is developed. This method allows to synthesize core–shell microcapsules of paraffin which have the shape of spheres or distorted spheres and are designed for the use as phase change materials. The microcapsules are formed in aqueous phase by the precipitation of *n*-eicosane together with modified polyhydromethylsiloxane from a common solvent which is miscible with aqueous media. The polysiloxane is modified by the attachment of silylvinyl and alkoxy functions before coemulsification with the paraffin. It also contains the Pt(0) Karstedt catalyst. The microcapsules formed by coemulsification are stabilized by the in situ cross-linking of the polysiloxane shell. The shell is additionally modified by the in situ generation of silanol groups which provide colloidal stabilization of microspheres in aqueous phase. Microcapsules were studied by DSC, SEM, optical polarized microscope, and by thermooptical analysis (TOA).

## Introduction

There has been a growing interest in phase change materials (PCM), which are converted from solid to liquid and from liquid to solid phase with a large heat effect. They are commonly used for thermoregulation, thermal energy storage, and cooling. Among many other applications they are used by textile industry for the thermoregulation of clothing [1–5]. In clothes, the PCM, which has a suitably matched phase change temperature, is soothing the effect of outside thermal peaks by the absorption and release of thermal energy. It gives the human body a feeling of thermal comfort and protects people working in adverse temperature conditions against thermal shocks. Paraffins, linear chain saturated hydrocarbons, with the melting and crystallization temperatures quite similar to the temperature of the human body (e.g., eicosane), are often used as PCM [6–8]. Importantly, they have a high latent heat of fusion which gives them high heat capacities. Their fusion temperature depends on the hydrocarbon chain length, so it may be easily adjusted to the needed temperature range. Moreover, paraffins are chemically and thermally stable and physiologically neutral.

Although PCMs are used in various forms, such as bulk materials, sheets, and particles dispersed in matrices, the most convenient form of their use in textile industry is core–shell microcapsules [9, 10]. Microcapsules can be easily dispersed in materials or attached to their surfaces. The rate of heat exchange with an encapsulated PCM is high due to its large surface to volume ratio. Moreover, microcapsules do not change the outer appearance of clothing and its characteristics, such as softness, breathability, and color. Melamine [11–15] or urea [16–18] resins cross-linked by formaldehyde are frequently used as the encapsulating material. Since there is a tendency to eliminate carcinogenic formaldehyde from the manufacturing of products used in contact with human body, our attention was turned to polysiloxanes as candidates for encapsulants for PCMs. These polymers are attractive because they are physiologically neutral, chemically and thermally stable, and are resistant to oxygen, water, and water vapor. They are sufficiently mechanically strong and elastic to withstand the volume change and deformation of PCMs during the phase change. The thermomechanical damage of the PCM microcapsule shell due to its rigidity may be a problem [18], and in some cases, soft segments are introduced into encapsulating polymer with purpose to prevent the shell breakage [19, 20].

Polysiloxane particles have been used as drug carriers for drug-controlled release [21, 22], carriers of metal catalysts [23, 24], dyes [25], and magnetic nanoparticles [26]. These particles are often named capsules but they do not have core–shell structure. They have form of microsphers in which other materials are dispersed. This type of particles does not fit for PCMs as the dispersion decreases paraffin thermal capacity. Organotrialkoxysilanes were used for the formation of polysilsesquioxane coating on particles by a sol–gel interfacial process [27, 28]. These coatings are rigid and may be cut or broken by sharp edges of crystals formed in the phase change process. Miniemulsion technique was recently used to encapsulate some materials with polysiloxanes. This method leads to the nano-sized core–shell particles [29, 30], which are not suitable for PCMs.

The encapsulation of a paraffin particle with polysiloxane to form a core–shell micron-sized structure is not an easy problem. Most of the encapsulation procedures are performed in aqueous systems. Polysiloxane monomers are soluble in paraffins and insoluble in water which makes the encapsulation of paraffin particles by an emulsion polymerization difficult. Coating of paraffin particles with a hydrophobic polysiloxane is also hindered because this polymer has a tendency to form a separate microphase in aqueous systems. Our attempts to use a polysiloxane with hydrophilic tetraalkylammonium chloride groups pendant to the polymer chain were unsuccessful as well. Unexpectedly we found that stable microcapsules with *n*-eicosane core and polysiloxane shell may be obtained when a modified polyhydromethylsiloxane is subjected to coemulsification with the paraffin in an aqueous system, with the in situ cross-linking of the encapsulant and with simultaneous additional modification of the formed particle shell. Our method is based on particular activity of the Karstedt catalyst (platinum Pt(0) complex with 1,3-divinyl-1,1,3,3-tetramethyldisiloxane) which may simultaneously act as the catalyst of four reactions involved in the encapsulation process, i.e., hydrosilylation [31], alcoholysis of hydrosilane [32], hydrolysis of SiH and SiH condensation with SiOH [33].

## Experimental

### Materials

Polyhydromethylsiloxane (PHMS) with trimethylsiloxane end groups was a product of ABCR Gmbh under name HMS 991 having viscosity 15–25 cSt corresponding to Mn = 1.5 × 10^3^ to 2.0 × 10^3^ g/mol; 1,3-divinyltetramethyldisiloxane (DVTMDS) with declared purity 97 % was provided also by ABCR poly[vinyl alcohol] (PVA) with declared purity 99.5 % and Mn 7.2 × 10^4^ g/mol was purchased from Polskie Odczynniki Chemiczne (POCH); isopropanol with declared purity 99.5 % was also obtained from POCH; tetrahydrofurane from CHEMPUR was p.a. grade dioxane with declared purity 99.8 % was purchased from CHEMPUR; *n*-eicosane with declared purity 99 % was from Alfa Aesar Gmbh. All these chemicals were used without additional purification; however, their purity was confirmed by the gas chromatography analysis. Platinum complex 20 wt% Pt(0) was kindly offered by Momentive Performance Materials Leverkusen.

### Physical methods

Thermal characteristics: differential scanning calorimeter (DSC) thermograms were registered with a DSC 2920 Temperature Modulated TA Instrument at the rate of temperature change 5 K/min and using ca. 5-mg samples. Thermogravimetric measurements were performed using a HIRES TGA 2950 Thermogravimetric Analyzer TA Instruments. Thermooptical studies were performed using a METTLER TOLEDO FP82HT HOT stage with control processor FP90.

Solid state ^29^Si NMR spectra were registered with a DSX 400 Bruker spectrometer. The spectra were acquired with cross-polarization, at 59.627 MHz applying 90-μs pulses, 6-s pulse delay, and 3-ms contact time, with samples in 4.0-mm zirconia rotors spinning at 8 kHz. The peak positions were referenced to the signal of Q_8_M_8_.

SEM images were taken with a JEOL JSH 5500 LV in high-vacuum mode at the accelerated voltage of 10 kV. Samples were coated with a fine gold layer, about 20 nm thick using ion coating JEOL JFC 1200 apparatus. Optical microscopy observations of microcapsules were done using microscope optical with polarizer Nicon Eclips E400 POL.

### Generation of microcapsules with *n*-eicosane core

The solution of 16.5 g of PHMS with 3.1 g of DVTMDS in 25 mL of tetrahydrofurane (THF) was heated to 45 °C and 0.025 g of a Pt(0) catalyst solution was introduced. An increase of viscosity of the solution was observed. The solution was stirred for 20 min at 45 °C and then introduced to a reactor in which the solution of *n*-eicosane 40 g in 110 mL of isopropanol was stirred at 45 °C. The resulting solution was stirred for additional 5 min and thermostated at 45 °C. Then the solution was quickly introduced to the 150 mL of water thermostated at 45 °C containing the 0.55 g of dissolved PVA. The mixture was homogenized for 25 s using a high-speed MPW-120 homogenizer set to 4,000 rpm. The formed emulsion was introduced to a 5-L reactor equipped with an anchor stirrer in which 1.85 L of the aqueous solution of the 6.55 g of PVA was stirred at 45 °C. Stirring was continued for 70 h after which microcapsules were separated by centrifugation. The isolated microcapsules were carefully washed with water to remove surfactant from their surfaces. Their shape did resemble distorted spheres and the yield of microcapsules with the linear size in the range between 2 and 12 μm was 70 %. Temperatures of fusion and crystallization were 37.4 and 30.1 °C respectively. The heat of fusion was 159 J/g.

## Results and discussion

### Preparation of microcapsules

**Fig. 1:**
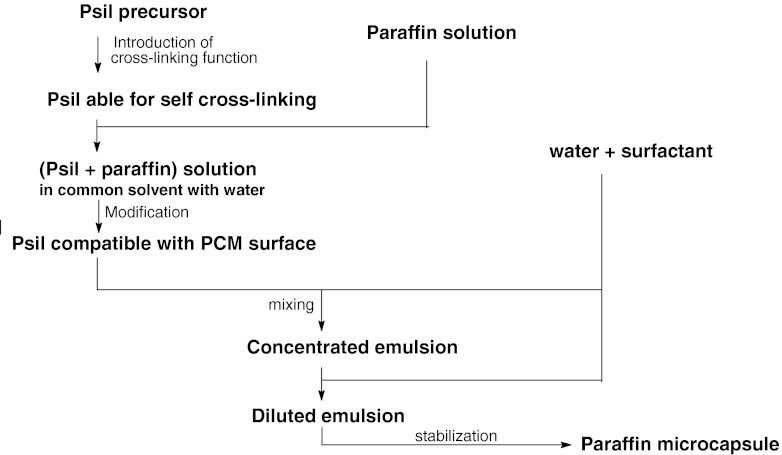
Scheme of the generation of paraffin microcapsules encapsulated in polysiloxane by coemulsification method

The general scheme of the synthesis of the paraffin core-polysiloxane shell microcapsules is shown in Fig. 1. The precursor of the encapsulating material is PHMS of molecular weight about 2,000 g/mol. This polymer is modified to gain the ability for selfcross-linking. For this purpose, it is subjected to hydrosilylation of DVTMDS cross-linker. The reaction, catalyzed by platinum Pt(0) complex (Karstedt catalyst), in its first stage proceeds according to Eq. 1, leading to PHMS with vinyl pendant groups bonded to polysiloxane chain by an ethylenedisiloxane bridge, Eq. 1.

1


The attachment of DVTMDS to the polysiloxane chain in the first step is necessary to prevent the penetration of this compound into the paraffin phase during the coemulsification and stabilization steps. However, the pendant vinyl groups may undergo subsequent hydrosilylation, which finally would results in the polymer cross-linking. This hydrosilylation is not desirable in the initial step, so at a suitable moment the reaction is slowed down by the dilution of the system with isopropanol solution of paraffin to give the time to perform further operations needed to form the polysiloxane coating on paraffin core before the cross-linking occurs.

In order to determine the moment at which the first step of the hydrosilylation process should be terminated, this reaction was parallelly monitored by the tracking of the variation of the viscosity of the reacting mixture. In the initial stage, when reaction 1 dominates, the viscosity increases slowly. The consecutive hydrosilylation of the second vinyl group is accompanied with a more rapid increase in the viscosity, which approaches infinity at gel point. At the half time of the hydrosilylation reaction needed to achieve gel point, i.e., well before the viscosity rapidly increases, the reacting mixture is added to solution containing the paraffin dissolved in a significant amount of isopropanol or in a mixture of isopropanol with dioxane or THF.

Then the hydrosilylation is effectively slowed down by the alcohol and by the dilution effect. The Pt(0) catalyst present in the reaction mixture promotes also the substitution of isopropoxy groups to the polymer chain, Eq. 2. Separate studies of this reaction revealed that several percent of SiH is transformed to SiO*i*Pr in this stage of the process. These isopropoxy groups improve the compatibility of the polysiloxane with the paraffin surface thus making easier the formation of the coating on paraffin particles. The using of isopropanol or its mixture with dioxane or THF is important as they dissolve well the polysiloxane-paraffin-catalyst mixture and at the same time they are miscible with water.2


The concentrated solution of the modified PHMS with paraffin is thermostated at a temperature above the paraffin melting point and is mixed, with a limited amount of aqueous solution of PVA used here as a surfactant. This operation is performed using a high-speed homogenizer, at a temperature above the melting point of paraffin. An emulsion of microdroplets of liquid paraffin embedded in polysiloxane is formed. The emulsion is unstable as the microcapsules are not fully formed and the encapsulant has not yet been cross-linked. The further physical and chemical processes are carried out after dilution of the emulsion by the PVA in water solution, having the same PVA concentration and the same temperature as the water used in the emulsification process.

The diluted emulsion is stirred for up to 70 h at a temperature higher than the paraffin melting point. A series of important chemical reactions occurs during this procedure, but it is worth noting that they must be preceded by the formation of the polysiloxane coating on paraffin microdroplets which takes place shortly after the dilution of emulsion. The chemical reactions which follow this physical process are shown in Eqs. 3–5. All of them are promoted by Pt(0) complex present in the polysiloxane shell.3
4
5


**Fig. 2:**
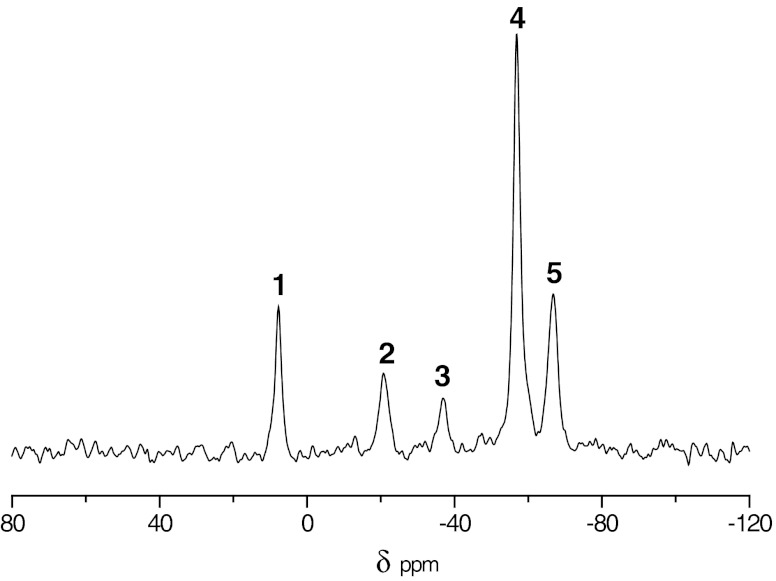
^29^Si MAS NMR spectrum of *n*-eicosane microcapsules encapsulated in polysiloxanes: *1*—(CH_2_)Me_2_ **Si**OSi + Me_3_ **Si**OSi; *2*—(CH_2_)Me**Si**(OSi)_2_; *3*—(H)Me**Si**(OSi)_2_; *4*—(HO)Me**Si**(OSi)_2_ + (iPrO)Me**Si**(OSi)_2_; *5*—Me**Si**(OSi)_3_

The cross-linking of the modified PHMS takes place in result of hydrosilylation of the vinyl group pendant to polysiloxane chain with SiH groups in another polymer chain. Thus, dialkyl disiloxane bridges are formed between polysiloxane chains, Eq. 3. The reaction is catalyzed by the Pt(0) complex which was introduced to PHMS in the initial hydrosilylation stage. This catalyst promotes also the hydrolytic cleavage of Si–H which leads to the generation of silanol groups on the polysiloxane chains, Eq. 4. These hydroxyl functions are very important because they make the microcapsules hydrophilic enhancing their dispersibility in aqueous systems. These groups are also useful for binding the microcapsules to a textile material surface. The silanol groups in the dehydrocondensation reaction with the SiH groups in polysiloxane catalyzed by the Pt(0) complex form siloxane bridges, Eq. 5. This reaction significantly contributes to the polysiloxane cross-linking and therefore makes shells of microcapsules stronger. Formally, the same bridges could be also formed in result of the homocondensation of silanol groups; however, this condensation does not proceed in the absence of any acidic, basic, or ionic substances, which are required for the catalysis of this reaction. All reactions 3–5 are promoted by Pt(0) complex located in the polysiloxanes shell, but the activity of the catalyst in this system is relatively low. Consequently, these reactions occur slowly which is advantageous because it provides enough time to shape shells of microcapsules before the encapsulant is cross-linked. The reactions which occur during the micropcapsule stabilization were monitored by ^29^Si MAS NMR. The NMR spectrum of the final product is shown in Fig. 2.

It should be mentioned that the Karstedt catalyst has been used so far mostly for the catalysis of hydrosilylation [31]. Its application for the catalysis of other reactions is very seldom, see references [32, 33]. In the process described here, the Pt(0) complex, besides hydrosilylation, catalyzes also three other above-mentioned reactions. All of them are very important for the successful synthesis of the microcapsule shells.

The isolation of microcapsules may be performed by sedimentation under gravitational forces or facilitated by centrifugation or by filtration. The density of the microcapsules is close to the density of water, which makes difficult their isolation by sedimentation. The presence of a submicron fraction of microparticles may pose a problem during the filtration. The microcapsules, after their isolation from the aqueous system, are subjected to freeze-drying. The yield of microcapsules was about 70 %. The optimal weight content of the polysiloxane coating was 33 %.

### Characterization of microcapsules

^29^Si MAS NMR spectra were analyzed with the purpose to obtain information on the chemical structure of the microcapsule shells. A spectrum of *n*-eicosan-containing microcapsules is shown in Fig. 2. The dominating peak at −57 ppm is that of silicon in the polymer chain bearing the hydroxyl group. A small signal at −59 ppm of silicon atom with bound izopropoxyl group is superimposed on the right wing of the silanol signal. The silicon in the polymer chain linking it to another chain by carbosiloxane and by siloxane bridges give resonances at −21 and −66 ppm, respectively. Rather, a small number of the unreacted SiH groups remains in the polymer giving signal at −37 ppm. The polysiloxane shells contain a great number of silanol groups which are present in the polymer bulk. The most important are those which are located on the shell surface because they make surface of microcapsules hydrophilic, which helps to redisperse them in aqueous systems after removal of surfactant.

**Fig. 3:**
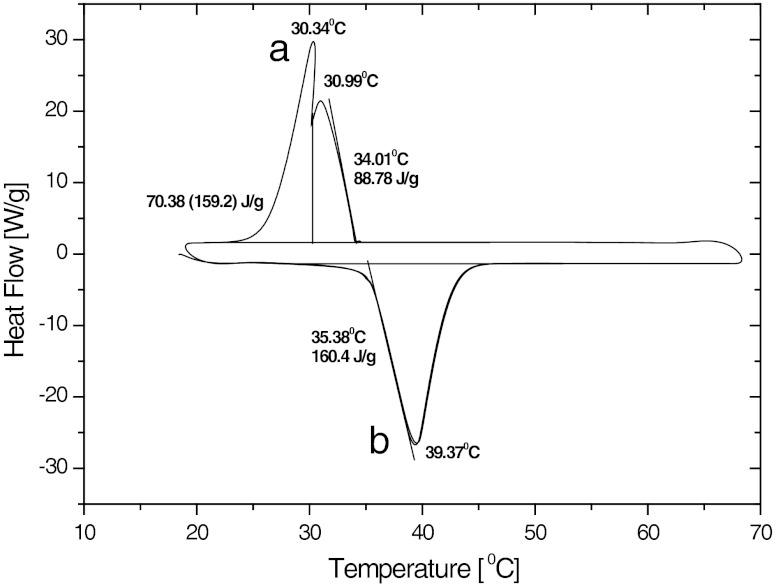
DSC thermograms of microcapsules of *n*-eicosane encapsulated in polysiloxane. *a* Cooling from melt, *b* Heating from crystals

Thermal behavior of microcapsules was studied by differential scanning calorimetry (DSC), and by thermooptical analysis. An example of the DSC thermograms corresponding to heating from the crystalline state and cooling from the molten *n*-eicosane in microcapsules is shown in Fig. 3. The endothermal peak of the PCM melting is of regular shape showing onset at 35 °C and maximum at 39 °C, which is somewhat above the melting temperature of pure *n*-eicosane, 36.2 °C. The difference results from the delay due to the rate of the temperature rise 5 K/min. At this rate, the phase change takes place within the range 35 to 43 °C The endothermal heat effect is 160 J/g which agrees well with the latent heat of melting of pure *n*-eicosane, 240 J/g, assuming that the 33 wt% of the microcapsule weight is occupied by polysiloxane shell. This conforms fairly well to the weight ratio of the polysiloxanes to paraffin used in the reaction. It should be mentioned that the DSC traces are not changed after 50 cycles of fusing and crystallization, which gives a proof of the thermal stability of the microcapsules.

The exothermal peak of crystallization of *n*-eicosane is splitted to two and ranges from 34 to 25 °C with maxima at 31 and 30 °C. The splitting comes from the polymorphism of *n*-eicosane [34].

Thermogravimetric studies showed that the onset of the *n*-eicosane microcapsule thermal decomposition is 156 °C. At this temperature, cracking of the silicone coating of microsphere begins and the maximum of this process is observed at 213 °C. Finally, almost all core is lost by evaporation at about 240 °C. Polysiloxane shell undergoes restructurization as the sample temperature is increased and relatively large amount of residue is generated (about 33 wt% at 900 °C). A high content of the residue is due to a high degree of cross-linking of the polysiloxane, which prevents its depolymerization and makes easier its transformation into the SiCO ceramics.

**Fig. 4:**
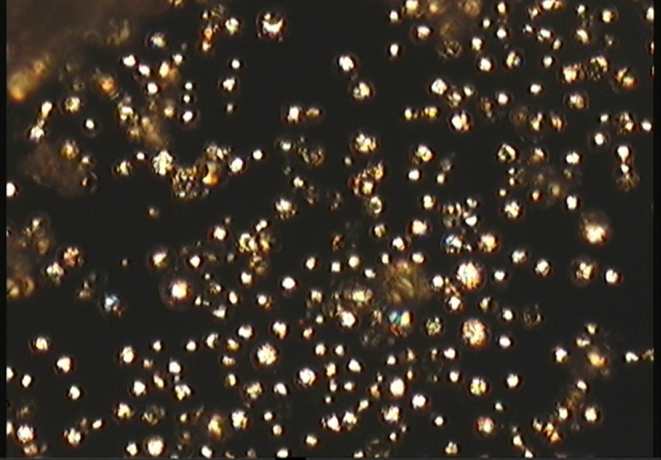
Photogram of microcapsules of n-eicosane coated with polysiloxane taken by optical microscope with the illumination of sample by polarized light and observation with cross-polarization

A microphotograph of *n*-eicosane microcapsules taken by an optical microscope with sample illuminated with polarized light and observation with cross-polarization is shown on Fig. 4. Isotropic shells of microcapsules built of polysiloxane are not visible on this photogram, but birefringent crystalline paraffin cores are visible. The microscope image of the microcapsules on a graphite surface does not change after many cycles of heating above the paraffin melting point and cooling to crystals. This confirms the thermal stability of the microcapsules. Any leakage would lead to the spreading out of the liquid paraffin over the surface due to the well-known ability of paraffins to interact with graphite surface forming monolayers [35].

**Fig. 5:**
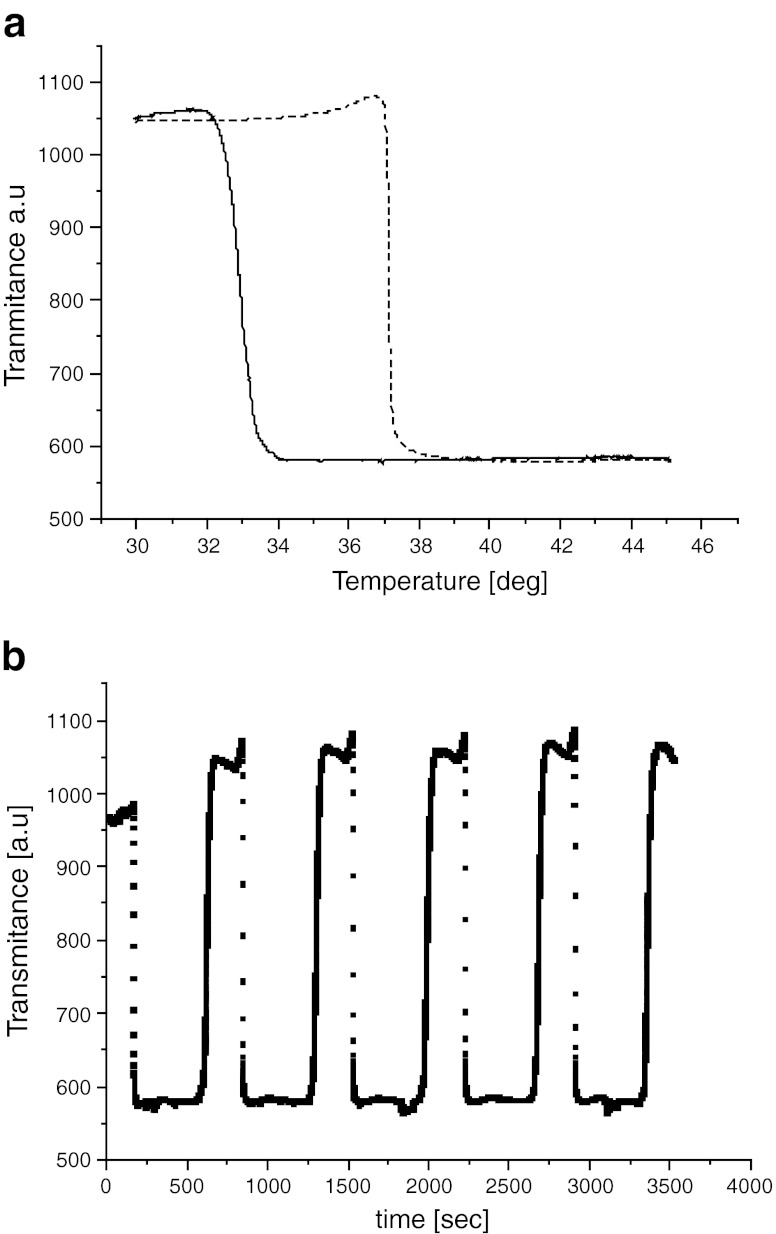
Thermal behavior of n-eicosane encapsulated in polysiloxane studied by thermooptical method (TOA): **a** *continuous line*—crystallization from melt, *dashed line*—melting from crystals; **b** Several cycles of cooling from melt and heating from crystals

A thermooptical method of studies of the thermal behavior of microcapsules is worked out. A sample of microcapsules is placed between two crossed polarizers. In the temperature range above the melting point of paraffin, both phases, i.e., the polysiloxane chain and the paraffin core, are isotropic, so very little light is allowed to go through this system and the transmittance is at a low level as shown in Fig. 5a. When microcapsules are subjected to cooling from the paraffin melt the paraffin crystallization begins. The crystallization is accompanied with light scattering that results in an apparent increase of the transmittance. On heating from the crystal state, the transmittance abruptly falls down thus indicating the paraffin melting. The temperatures of fusion and crystallization of PCM may be readily determined from the transmittance traces, Fig. 5a. They agree well with the corresponding temperatures of melting and crystallization found by DSC, when both experiments are performed with the same heating and cooling rate. The thermooptical method may be used for the study of the behavior of microcapsules in multiple cycles of heating from crystal to liquid state and cooling from liquid to crystal. Figure 5b shows traces of transmittance intensity changes for several cooling and heating cycles. These traces do not change their shapes, which can be considered as another proof of the thermal stability of the microcapsules.

**Fig. 6:**
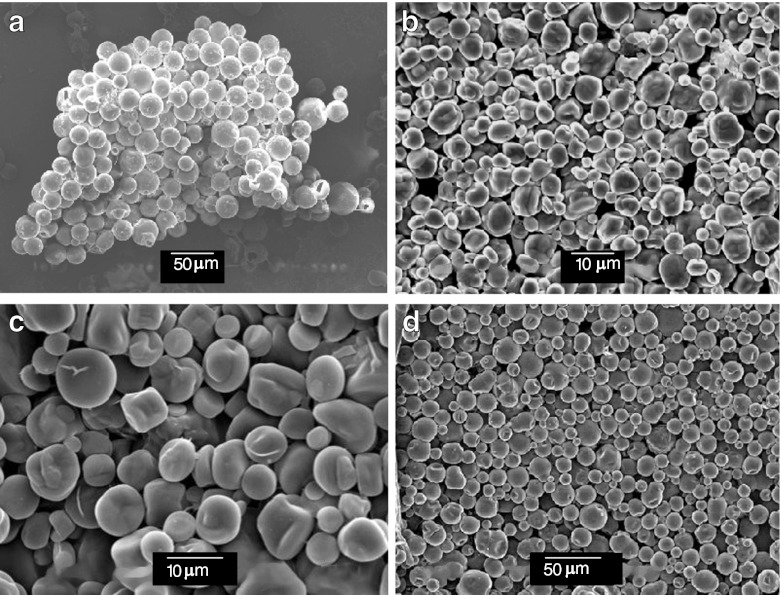
SEM images of microcapsules with n-eicosane core and polysiloxane shell obtained using conditions of synthesis and characteristics which are mentioned in Table 1: **a** — exp. I, **b** — exp. II, **c** — exp. III, **d** — exp. IV

Microcapsules are studied also by scanning electron microscopy (SEM). Examples of SEM images of encapsulated *n*-eicosane are shown in Fig. 6. Microcapsules have spherical or distorted spherical shapes. Their mean sizes estimated from the SEM images are shown in Table 1. They ranged from 5 to 22 μm. The average size and shape of the microcapsules depend on conditions of their formation. Some parameters of syntheses and characteristics of microcapsules obtained in various experiments are given in Table 1. A very important parameter is the time of the initial hydrosilylation step, i.e., time in which the DVTMDS cross-linker should be added to the PHMS chain. If this time period is too long, the vinyl group pendant to the polysiloxane chain enters hydrosilylation that results in a branching of PHMS and in an increase in molar mass of the polymer. As a result, the viscosity of PHMS is high when it precipitates in aqueous phase. The process described above leads to a distortion of microcapsules from their spherical shape as it is shown in Fig. 6bc. However, these distorted microcapsules, although not spherical, may have good thermal properties and may be stable, as it is shown in Figs. 3 and 5b. On the other hand, the time of the initial hydrosilylation cannot be too short, because an unreacted DVTMDS may penetrate into paraffin and deteriorate thermal properties of the microcapsules. The time of the initial hydrosilylation depends on the catalyst activity, which is sensitive to contaminations of used chemicals. This time was chosen as the half time of the hydrosilylation needed to achieve gel point and it was determined in a separate experiment for each synthesis.

**Table 1:** Synthesis of paraffin encapsulated in polysiloxane

Number	PCM (quantity) g	Solvent	PCM/solvent, g/mL	Catalyst Pt/capsule^a^, mol/g	Time of cross-linker addition, min	Heat of melt, J/g	Melt. temp. (range), °C	Cryst. temp. (range), °C	Mean size of capsule, μm
I	*n*-eicosane (11)	Isoprop.	0.37	9.7 × 10^−7^	4	139	37.4 (35–42)	30 (35–23)	22.9
II	*n*-eicosane (20)	Isoprop.	0.33	7.8 × 10^−7^	10	158	37.4 (35–42)	30 (34–22)	6.3
III	*n*-eicosane (40)	Isoprop. + THF 5:1 *v*/*v*	0.30	4.3 × 10^−7^	20	159	39.1 (35–42)	30 (34–22)	5.9
IV	*n*-eicosane (240)	Isoprop. + dioxane 1:1 *v*/*v*	0.55	3.2 × 10^−7^	10	160	39.4 (35–43)	31 (34–25)	10.1

Parameters were: polysiloxanes to paraffin *w*/*w* ratio 0.5, cross-linker (DVTMDS) to precursor polymer (PHMS) *w*/*w* ratio 0.19, mixing (homogenization in water): time 0.5 min., temperature 45 °C, water to capsule ratio 2.5 mL/g, surfactant (PVA) concentration in water 3.6 g/L, stabilization: water to capsule milliliters per gram ratio 30–35 mL/g, time 30–70 h, temp. 45–60 °C. PVA concentration is the same as in the mixing operation

^a^PHMS + DVTMDS + *n*-eicosane

## Conclusions

The coemulsification in aqueous phase of *n*-eicosane with polyhydromethylsiloxane having pendant vinyl groups may lead to the formation of core–shell microparticles in which the paraffin is embedded in a polysiloxane shell. These particles are in situ stabilized by the polysiloxane cross-linking using the hydrosilylation process catalyzed by Pt(0) complex (Karstedt catalyst). This catalyst promotes also the competitive formation of silanol groups on the polysiloxane and their partial condensation with the SiH groups which additionally crosslinks the polysiloxanes shell.

The coemulsification is carried out in the temperature above the paraffin melting point using isopropanol or its mixture with THF or dioxane as common solvents for the paraffin-polysiloxane-catalyst system, which are miscible with water. These core–shell particles may serve as PCM microcapsules. They have a good thermal characteristic and withstand 50 fusion-crystallization cycles test.
